# Correlation Between Ki-67 Index, World Health Organization Grade and Patient Survival in Glial Tumors With Astrocytic Differentiation

**DOI:** 10.7759/cureus.1396

**Published:** 2017-06-26

**Authors:** George S Stoyanov, Deyan L Dzhenkov, Martina Kitanova, Ivan S Donev, Peter Ghenev

**Affiliations:** 1 Department of General and Clinical Pathology, Forensic Medicine and Deontology, Faculty of Medicine, Medical University – Varna “Prof. Dr. Paraskev Stoyanov”, Varna, Bulgaria; 2 Clinic of Oncology, St. Marina University Hospital Varna

**Keywords:** gbm, ihc, ki-67, survival analysis, automated cell counting

## Abstract

Background

Glioblastoma multiforme (GBM) is a class IV astrocytic tumor, the most malignant of the four groups of World Health Organization (WHO) tumors with astrocytic differentiation.

Aim

The aim of this study was to estab­lish whether a correlation exists between the Ki-67 index of tumors with astrocytic differentiation, WHO grade, and patient survival.

Materials and methods

A retrospective non-clinical approach to patient selection was chosen for the aim of the study. A total of 47 patients diagnosed and treated for CNS tumors with astrocytic differentiation in the St. Marina University Hospital, Varna, Bulgaria, from September 2012 to July 2016 were retrospectively included into the study cohort. The cases were tested for their immunohistochemistry (IHC) reaction with Ki-67 after their original Hematoxylin and Eosin and IHC slides were reviewed by a single author and blind coded. The Ki-67 positivity index of the nuclei was estimated after digitalization of the slides and calculated by the ImmunoRatio automated count­ing tool. The individual Ki-67 index and patient survival of each case were statistically compared.

Results

The histopathological groups, after the blind Ki-67 index automated calculation was carried out, revealed no WHO grade I, two WHO grade II samples, four WHO grade III samples and 41 WHO grade IV cases, and these were included in the analysis. The two samples of WHO grade II astrocytic tumors had a mean Ki-67 index of 25%; however, they comprised tumors with an individual index of 43% and 7%, both individual values with a highly unlikely index for this group. The four samples of WHO grade III had a mean Ki-67 index of 4%, standard deviation ±2.16 (p>0.05), with the lowest index being 1% and the highest one being 6%. Both WHO grade II and III did not include enough samples to allow for a proper statistical analysis of patient survival. The 41 GBM cases had a mean Ki-67 index of 17.34%, standard deviation ±10.79 (p>0.05). Statistical analysis of the Ki-67 index divid­ed dichotomously into two groups and patient survival revealed that cases with a high Ki-67 index had no significant difference in survival when compared to those with low expression.

Conclusions

Based on the reported results, the mean Ki-67 percentage of positive nuclei in GBM tumor sam­ples cannot be used to estimate the survival of patients. However, Ki-67 remains a valuable IHC pathological tool.

## Introduction

The World Health Organization (WHO) guidelines classify central nervous system (CNS) astrocytic tumors into four malignant groups based on their histopathological hallmarks: grade I astrocytoma, with the best patient prognosis and least aggressive course of progression; grade II astrocytoma, a more aggressive course and less favorable patient prognosis; grade III astrocytoma or anaplastic astrocytoma (AA), with an overall bad patient prognosis; and grade IV astrocytoma, referred to as glioblastoma multiforme (GBM), with highly atypical cells, sometimes referred to as monstrous, rapid progression and the worst patient prognosis of the IV classes [1].

High grade astrocytic tumors of the CNS, referred to as grades III and especially IV, are considered the most malignant entries in oncology. These tumors are rarely diagnosed in their early stages, are hard to treat surgically and often pose a challenge for histopathological diagnosis due to their tendency to be highly heterogenic and mimic other tumor types [2]. The medical treatment strategies, although some of them are aimed at specific cell mutations, are not as effective as in other oncological entries and the five-year patient survival rate in some classes is less than 3% [3].

Lower grade astrocytic tumors have a high recurrence rate and often progress to more malignant lesions—WHO grades III and IV. However, unlike other oncological entries, with astrocytic tumors, the most malignant form—grade IV—is the most commonly diagnosed entry, with some studies estimating that nearly 90% of CNS astrocytic tumors are classified as GBM [4].

In some oncological entries, the immunohistochemistry (IHC) marker for cell proliferation, Ki-67, has been shown to correlate with and even estimate the tumor malignancy class and patient prognosis [5-7]. The design of this study was aimed first at establishing an easy, quick, and reliable way for determining the Ki-67 index of tumor samples, which can be applied in the everyday pathological practice, without the need for time-consuming methods of cell counting, a high-end computer configuration, or software skills. The second part of the study design was aimed at establishing the Ki-67 index of astrocytic tumor samples and determining whether the index can be used as a discriminating factor when determining the WHO grade of the tumor sample and establishing the relevance of the Ki-67 index to patient survival.

## Materials and methods

Patient selection

A retrospective non-clinical approach to patient selection was chosen for the study. A total of 47 patients diagnosed and treated for CNS tumors with astrocytic differentiation in the St. Marina University Hospital, Varna, Bulgaria, from September 2012 through July 2016, were retrospectively included in the study cohort. Data regarding clinical representation, histopathological diagnosis, and patient survival post diagnosis were all included in the study from the central digital hospital database. A number of cases diagnosed and treated in the set timeframe were not included in the study due to primary diagnosis or treatment in another medical institution, medical documentation that could not be retrieved, or insufficient tumor tissue material in the paraffin fixed tissue blocks, on which further histopathological investigations could not be carried out.

Histopathological review and slide preparation

The histopathological samples of astrocytic tumors were collected from the central pathological archive of a single tertiary health center—St. Marina University Hospital, Varna, Bulgaria. IHC staining with Ki-67 was performed on the Dako Autostainer Link 48 (Dako/Agilent Technologies, CA, USA) using ready-to-use catalogue Dako primary and secondary antibodies with chromogen (Dako/Agilent Technologies, CA, USA). Digital scans of the IHC slides were taken on a Leika Aperio AT2 automated slide scanner (Leica Biosystems Inc., IL, USA) (original magnification of digital slides 400x) and archived for analysis.

A single-pathologist review was carried out for all cases on the original Haemotoxylin and Eosin (H&E) and IHC slides used from the primary diagnosis. This evaded unintentional blinding of the result from cases misinterpreted as astrocytic tumors or WHO graded improperly, but falling into another category.

Once reviewed, the IHC slides were blind coded by one of the authors to evade suggestive selections, ensuring a blind histopathological analysis of Ki-67 expression.

Cell counting method

The cell counting tool selected for the Ki-67 indexing was the automated cell counter for digital histology slides ImmunoRatio (a free web application), as it is available as a free tool with both browser-based analysis and an installable plugin for the open source ImageJ (NIH, Bethesda, Maryland) image analysis software and does not require a high-end computer configuration [8-9]. The automated cell counting in the app is based on color deconvolution, targeted specifically at nuclear expression IHC images and has been shown to have a cell counting error comparable with manual real-time microscopy counting, manual cell counting on digital slides and other automated cell counting software, available in more constrict circumstances or needing greater software skill of the operator and higher-end computer configuration [8-9].

**Figure 1:**
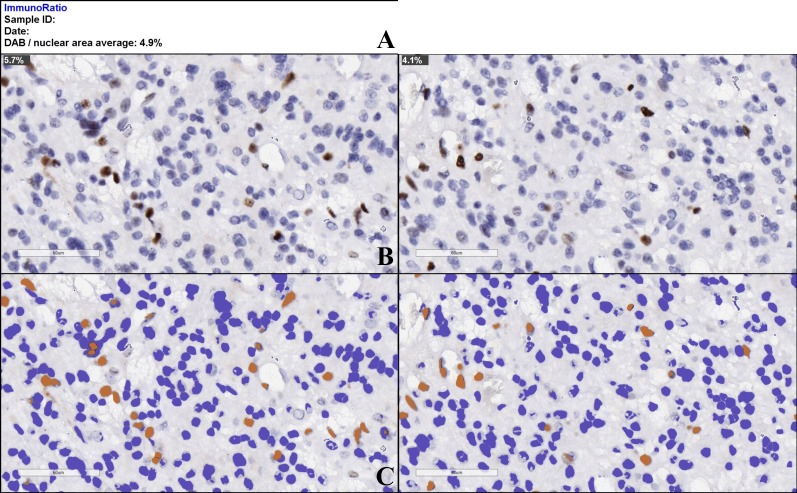
ImmunoRatio automated cell counting tool stock results–Ki-67 index estimation (DAB indicates the mean average percentage of positive nuclei for both analyzed images). A – estimation of average Ki-67 index for the set of uploaded images, B – original uploaded image with individual Ki-67 index, C – falsely colored images, automatically generated by the algorithm for nuclear expression estimation. DAB - 3,3′-Diaminobenzidine.

All cases were evaluated for their Ki-67 index by a single author. The criteria for Ki-67 indexing were a human selection of the most positive area and the most negative area of each slide and using the ImmunoRatio built-in capabilities to establish the mean coefficient of Ki-67 positivity for each individual area and the mean of the two (Figure 1). Additional criteria included exclusion of highly vascular areas in the selected fields, as vessel endothelial cells could possibly falsely increase the Ki-67 index, and absence of a pseudopalisadic necrosis formation or surgery-induced necrosis, to ensure the maximum number of cells per viewing field possible.

Statistical approach

Statistical analysis was carried out with IBM SPSS Statistics V23 (IBM, NY, USA) using the descriptive statistical approach. Categorical features were summarized with frequencies and percentages. Survival curves were estimated by the Kaplan-Meier method with differences assessed by the log-rank test. Although our study did not include enough cases to compare different subgroups, the hazard ratios and corresponding 95% confidence intervals were calculated by Cox regression models. A two-tailed test was used to determine statistical significance and a p-value of <0.05 was considered significant.

## Results

The histopathological groups revealed after the blind Ki-67 index automated calculation showed no WHO grade I, two WHO grade II samples, four WHO grade III AA samples and 41 WHO grade IV tumors, and these were included in the analysis. The individual statistical values of the three separate WHO grade groups included into the analysis are as follows:

WHO grade II

The two samples of WHO grade II astrocytic tumors had a mean Ki-67 index of 25%; however, they comprised tumors with an individual index of 43% and 7%, both highly unlikely values for this grade. As the data selection was too small and the two separate tumors had a great difference in their Ki-67 index, this did not allow for a proper further statistical analysis of patient survival.

WHO grade III

**Figure 2:**
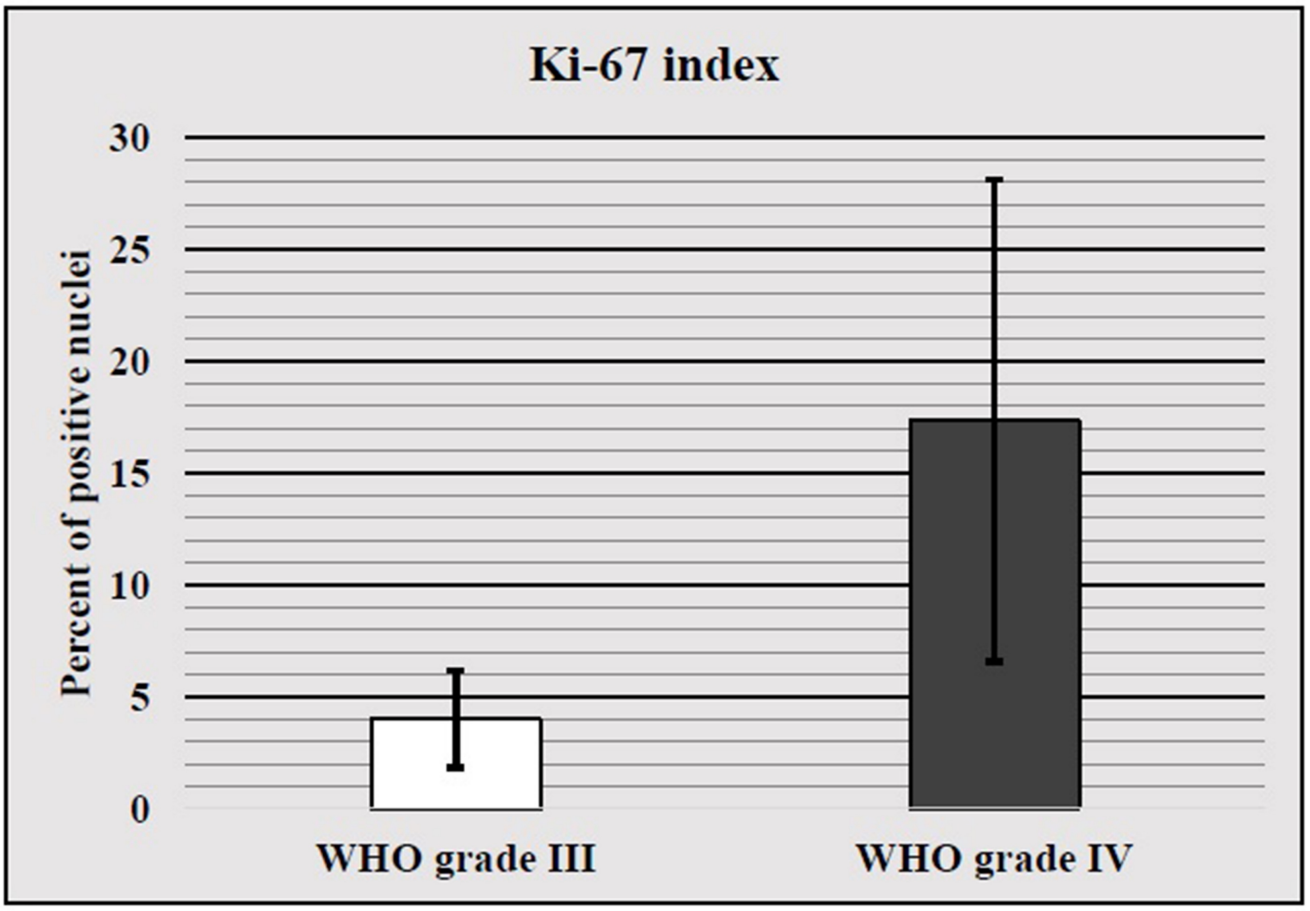
WHO III and IV mean Ki-67 index with standard deviation.

The four samples of WHO grade III AA had a mean Ki-67 index of 4%, standard deviation ±2.16 (p>0.05), with the lowest index being 1% and the highest one being 6% (Figure 2). As with WHO grade II astrocytoma, the data selection was too small and did not allow for a proper further statistical analysis of patient survival.

WHO grade IV

**Figure 3:**
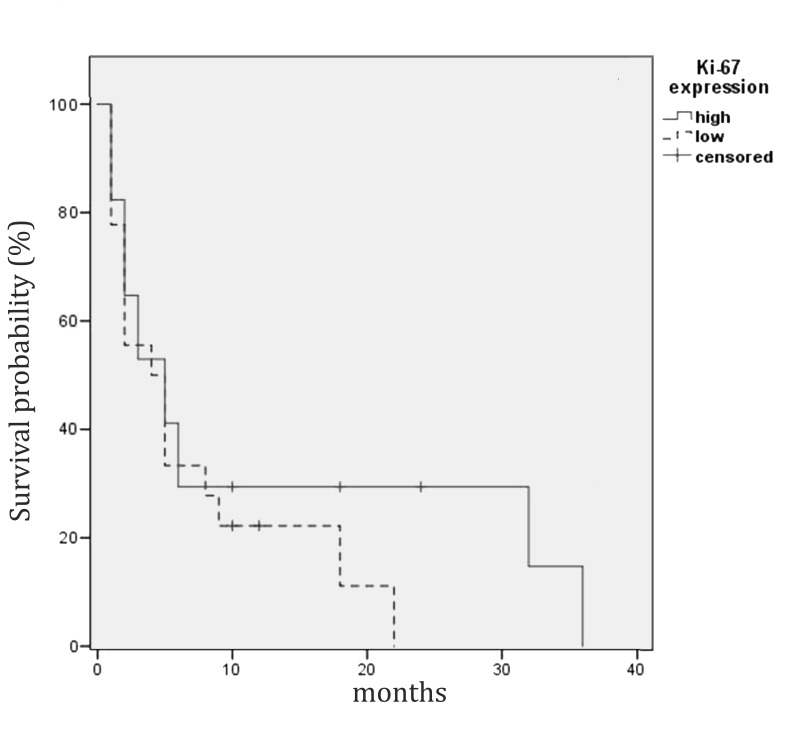
Kaplan-Meier estimates of WHO grade IV astrocytic tumor patients' survival by Ki-67 index levels (dichotomized by median value to high and low expression levels). There was no difference in the median survival between the two groups.

The 41 samples of WHO grade IV astrocytic tumors had a mean Ki-67 index of 17.34%, standard deviation ±10.79 (p>0.05), with the lowest index being 2% and the highest one being 46% (Figure 2). Statistical analysis of the Ki-67 index divided dichotomously into two groups (low and high) and patient survival revealed that cases with a high Ki-67 index had no significant difference in survival when compared to those with low expression (Figure 3).

Based on the reported results, there is no clear correlation between the Ki-67 index of an astrocytic tumor sample and the histopathologically determined WHO grade and patient survival. Therefore, the reported figures do not represent any statistical significance whatsoever (p>0.05).

## Discussion

Our results show no statistically significant data suggesting a correlation between the WHO grade and patient survival when compared to the Ki-67 index. This data contradicts related studies which describe such a correlation [10-17]. However, some of these studies do not state the means of calculating the Ki-67 index [11]. Other research teams state that the estimation was carried on pathologist interpretation only, excluding a standardized approach to Ki-67 index estimation [14, 17]. In some of these studies, a very strong statistical correlation is declared with a very low p-value, whilst at the same time the standard deviations of different groups significantly overlap one another [13, 16-17].

Only one study states that whilst whole slide counting for Ki-97 index correlates with WHO grade and patient survival, it does not correlate when the same slides are evaluated on most positive sections, underlining the lack of everyday pathological significance [18].

A third set of research articles, focused on everyday pathological diagnostics, are in support of our findings, stating overlap of Ki-67 index in-between WHO grades and no statistically significant correlation with patient survival [19-21].

A common feature, however, is the lack of comparable results for the high and low index groups in-between studies, with different teams using 5%, 10% or 20% margin for a cutoff value. This not only yields all results incomparable, but also contradictory to one another [14, 17, 21].

Unlike other, much more established markers in astrocytic tumors, such as the presence of an IDH1/2 mutations, 1p/19q translocation and MGMT mutations, Ki-67 does not seem to be of viable significance in the everyday pathological verification of these oncological entries [1, 3, 22-24].

Ki-67, however, remains a valuable IHC tool in pathology and its role as established in other malignancies remains unquestioned.

## Conclusions

Based on the reported results, the mean Ki-67 percentage of positive nuclei in an astrocytic tumor sample cannot be used to properly determine the WHO grade and estimate the survival of the patients, despite some reports in literature stating otherwise. These reports cannot be taken into account due to their inapplicability into everyday pathology practice, their irreplaceable results, and the lack of standardized estimation and statistical approaches.

At this point, the only sure way to determine the histopathological WHO grade remains the pathohistological evaluation of the H&E stained tumor sample.

Therefore, the only verified prognostic markers for astrocytic tumors remain the IDH1/2 mutation, 1p/19q translocation, and the presence of an MGMT mutation.
